# 

**Published:** 1899-06

**Authors:** 


### BUFFALO MEDICAL JOURNAL

**Established 1845 by AUSTIN FLINT, M. D.**

**EDITORS:**

THOS. LOTHROP, M. D. WM. WARREN POTTER, M. D.

**ASSOCIATE EDITORS:**

WILLIAM C. KRAUSS, M. D.
JAMES WRIGHT PUTNAM, M. D.
JOHN A. MILLER, PH. D.
JOHN PARMENTER, M. D.
ERNEST WENDE, M. D.
ALVIN A. HUBBELL, M. D.

LIBRARY
SURGEON GENERAL'S OFFICE
MAY-[illegible]-1899

SCRIPTA FERUNT ANNOS

EUROPEAN AGENCY, J. B. BAILLIERE & FILS, 19 Rue Hautefeuille, Paris.
*Subscription, if paid in advance, $2.00; otherwise, $2.50. Foreign subscription, $2.50.*
Copyright 1899, by WILLIAM WARREN POTTER, M. D.
Entered at the Post Office at Buffalo, N. Y., as second-class mail matter.

A. T. BROWN PRINTING HOUSE, BUFFALO, N. Y.

**Table of Contents on Page V.**

